# Exploring the relationship between problematic internet use and emotion regulation skills in tunisian medical students

**DOI:** 10.1192/j.eurpsy.2021.1533

**Published:** 2021-08-13

**Authors:** A. Rhouma, B. Saguem, S. Ben Nasr

**Affiliations:** Psychiatry, farhat hached hospital, sousse, Tunisia

**Keywords:** Medical Students, Problematic Internet use, emotion regulation skills

## Abstract

**Introduction:**

There is paucity of researches addressing the relationship between individuals struggling to identify, express and communicate their emotions and problematic internet use, especially among medical students.

**Objectives:**

To assess problematic internet use in Tunisian medical students and to address its relationship with emotion regulation skills.

**Methods:**

First to fifth-year undergraduate medical students registered in the medical school of Sousse, Tunisia, were offered to answer an online questionnaire survey, involving sociodemographic and clinical data, Internet Addiction Test (IAT) and Difficulties in Emotion Regulation Scale (DERS).

**Results:**

A total of 175 medical students participated in the study with a median age of 22 (20-23) years and a gender ratio of 0.3. Median score of IAT was 40 (30-48). Twenty-four percent of medical students (n=42) reported problematic Internet use. Higher scores of IAT were significantly associated with the perception of an unsatisfactory relationship with parents, not having a leisure activity, family history of psychiatric disorders, personal health conditions and regular alcohol consumption. Scores of IAT were strongly and positively correlated with the following DERS subscores: Non acceptance of emotional responses (r=0.328**), Difficulties engaging in goal directed behaviors (r=0.366**), Impulse control difficulties (r=0.238**), Limited access to emotional regulation strategies (r=0.311**), and Lack of emotional clarity (r=0.311**).

**Conclusions:**

Problematic internet use seems to emerge as part of a cluster of symptoms related to ineffective emotion regulation skills. Hence, training for affective regulation abilities appears strategically useful in the control of Internet use.

